# Supplementary material for: Variations in grain yield and nutrient status of different maize cultivars by application of zinc sulfate
Source: PLoS One. 2024 Mar 8;19(3):e0295391. doi: 10.1371/journal.pone.0295391 (PMC10923429; doi:10.1371/journal.pone.0295391)
Supplement: S1 Fig — (DOCX) [file pone.0295391.s001.docx]

**
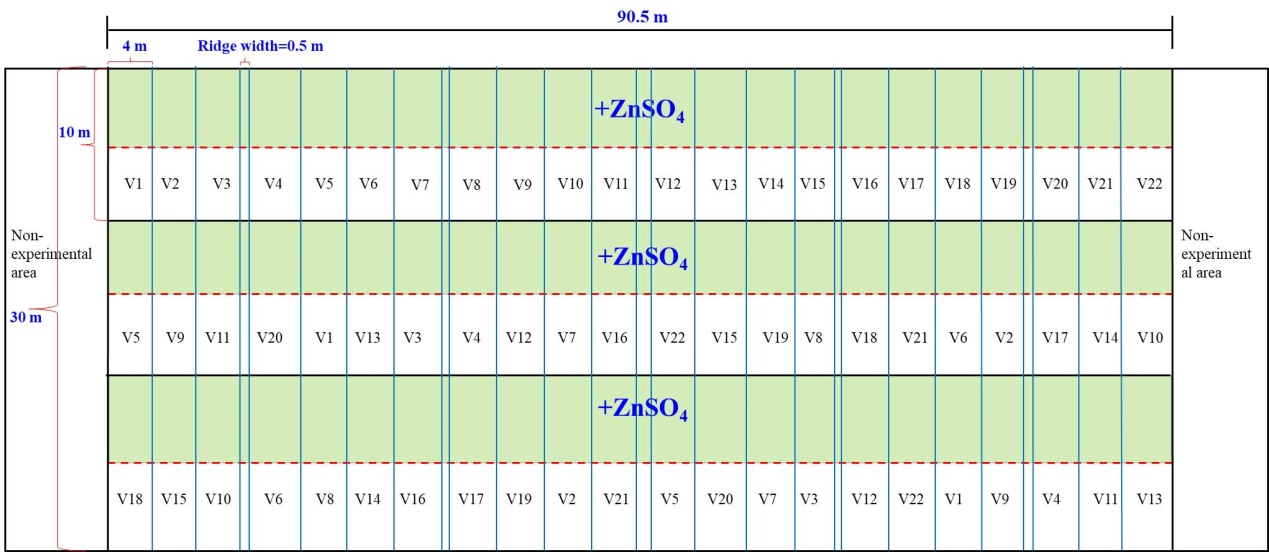
**

**Figure S1 Field layout of the split-plot design experiment.** V1 to V22 represent different maize varieties. The green area represents applied zinc fertilizer, while the white area represents applied distilled water (control).
