# Supplementary material for: Variations in grain yield and nutrient status of different maize cultivars by application of zinc sulfate
Source: PLoS One. 2024 Mar 8;19(3):e0295391. doi: 10.1371/journal.pone.0295391 (PMC10923429; doi:10.1371/journal.pone.0295391)
Supplement: S1 Table — (DOCX) [file pone.0295391.s002.docx]

**Table S1** The name for selected 22 modern maize cultivars.

| Maize type | Name of cultivar |
| --- | --- |
| Zn-deficiency sensitive | Xianyu048; Lianchuang808; Songyu619; Bangyu339; Denghai685; Shihai916 |
| Zn-deficiency non-sensitive | Luodan9; Hualiang78; Xianyu1140; Shengyu18; Xianyu335; Cunyu10; Lianchuang3; Ningyu614; Luomei1; Nonghua816; Xianyu047; Lianchuang825 |
| Zn-deficiency resistant | Jiamei1; Tunyu061; Xianyu045; Fanyu298 |
