# Supplementary material for: Variations in grain yield and nutrient status of different maize cultivars by application of zinc sulfate
Source: PLoS One. 2024 Mar 8;19(3):e0295391. doi: 10.1371/journal.pone.0295391 (PMC10923429; doi:10.1371/journal.pone.0295391)
Supplement: Attachment — Submitted filename: 20231104 Reponse to reviewers comments (PONE-D-23-07689R1).docx [file pone.0295391.s006.docx]

**Dear Editors and Reviewers,**

Thank you very much for your advice and comments are relates to our manuscript. Your comments are very useful indeed toward the improvement of this draft. The modifications made in the manuscript are marked in revised file. Our point-by-point responses to the comments of reviewers’ are included below as inserts marked in blue.

Thank you very much for considering our manuscript for publication in *PLos One*. I am indeed grateful for your time, and look forward to hearing from you soon!

Best wishes,

Dr. Juan Xin

Zhengzhou Normal University, Zhengzhou, 450044, China.

E-mail: xinjuan0707@163.com

----------------------------------------

**Journal Requirements:**

Please review your reference list to ensure that it is complete and correct. If you have cited papers that have been retracted, please include the rationale for doing so in the manuscript text, or remove these references and replace them with relevant current references. Any changes to the reference list should be mentioned in the rebuttal letter that accompanies your revised manuscript. If you need to cite a retracted article, indicate the article’s retracted status in the References list and also include a citation and full reference for the retraction notice.

Response (R): Thank you for providing us with a valuable revising opportunity. We have carefully improved our references.

**Additional Editor Comments:**

The manuscript was substantially improved. However, it still requires some adjustments to ensure the quality required.

The reviewers indicate the following points:
1. Insert the size of the experiment as well as the dimension of the elementary plot.

R: We have revised this figure (Fig. S1), inserting the dimension of the elementary plot.

Figure S1 Field layout of the split-plot design experiment. V1 to V22 represent different maize varieties. The green area represents applied zinc fertilizer, while the white area represents applied distilled water (control).

2. LINE 163-164: This sentence doesn't have a clear meaning.

R: Yes, ‘All methods in this article were carried out in accordance with relevant guidelines in the method section’ this sentence doesn’t have a clear meaning. We deleted the sentence after consideration, thank you.

3. LINE 228 "....yields and grain numbers of different maize. " What grain numbers refer to in this sentence here and after that?

R: ‘Grain numbers’ refer to ‘No. of grains per panicle’ in this paper, we have supplemented this phrase.

4. Check the English language of the manuscript (please see corrections in the attached file).

R: Thank you for your help in improving the language of our paper, and we have revised them as your suggestion.

5. Table 1 repeats the information described in the manuscript's text (please choose one option: Table or text).

R: We deleted the Table 1, thank you.

6. Please check the references to homogenize and adjust to the requested style.

R: We have checked all the references according to the journal's requirements.
